# “Controlled by Female Hormones”: A Qualitative Interview Study of Swedish Women’s Experiences of Gender-Specific Aspects of Life With ADHD

**DOI:** 10.1177/10870547261427555

**Published:** 2026-03-26

**Authors:** Julia McTaggart, Lisa B Thorell, Charlotte Borg Skoglund, Niklas Envall, Helena Kopp Kallner

**Affiliations:** 1 Danderyd Hospital, Stockholm, Sweden; 2 Karolinska Institutet, Stockholm, Sweden; 3 Smart Psykiatri AB, Stockholm, Sweden; 4 Uppsala University, Sweden; 5 Dalarna University, Falun, Sweden

**Keywords:** ADHD, qualitative research, hormones, adult

## Abstract

**Objective:**

There is limited knowledge about how female specific factors such as fluctuating sex hormones influence symptom display and health-related conditions that are unique to, or more prevalent in females with ADHD. This study aims to investigate how women of reproductive age with ADHD experience their ADHD symptoms and well-being in relation to hormonal fluctuations, and secondly, how they perceive hormonal and reproductive counseling in healthcare.

**Method:**

Semi-structured interviews were conducted with 14 women with ADHD and analyzed using inductive thematic analysis.

**Results:**

Three main themes emerged from the analysis; (1) Controlled by female hormones, (2) Frustration with lack of knowledge/understanding, and (3) Living with ADHD and comorbidities. Many women have experienced challenges and fluctuations related to hormonal changes during their menstrual cycles and in different stages of life. Using hormones to stabilize mood and impulsivity and adjusting stimulant doses were suggested as potential solutions. Participants expressed frustration about the lack of knowledge, interest, and understanding from healthcare professionals regarding ADHD and how hormones influenced symptoms of ADHD and comorbidities in women.

**Conclusion:**

This qualitative study highlights the impact of cyclical hormonal fluctuations on daily functioning across the menstrual cycle. Specifically, participants reported cyclic patterns of high energy and productivity related to ovulation followed by low energy and difficulty managing tasks in the premenstrual week. This aligns with clinical experience, anecdotal evidence, and limited literature that suggest that women with ADHD may be particularly vulnerable to hormonal fluctuations. Our findings suggest that especially the premenstrual phase is a challenging time for women with ADHD. Our results emphasize the need for healthcare professionals to improve their understanding of the role sex hormones and the menstrual cycle play in female ADHD. The potential effect of adjusting ADHD medication dosage and introducing hormonal treatment for premenstrual dysphoric disorder (PMDD) in women with ADHD should be further explored.

## Introduction

ADHD is a neurodevelopmental disorder characterized by symptoms of inattention, hyperactivity, and impulsivity. Across the lifespan, ADHD is associated with a plethora of negative psychosocial and health outcomes, such as low quality of life, academic underachievement, unemployment, teenage pregnancies, relationship problems, sleep problems, addiction, and delinquency (Faraone et al., 2021). Historically, most research has focused on boys and men leading to several knowledge gaps regarding how biological sex differences, fluctuating sex hormones, and the use of hormonal contraceptives influence ADHD symptoms and comorbid conditions (Martin, 2024; Young et al., 2020). Previous research demonstrated that females are diagnosed with ADHD approximately 4 years later than males (Skoglund et al., 2024). As a result, boys often receive earlier intervention during critical developmental periods. Females tend to present with more severe impairment at diagnosis, report higher healthcare utilization, and are more frequently prescribed pharmacotherapy for comorbid conditions (Skoglund et al., 2024). The limited understanding of female ADHD, especially the role of sex-specific factors, may contribute to delayed diagnosis and increased psychiatric and somatic comorbidity and healthcare utilization in girls and women with ADHD.

Emerging evidence indicates that many psychiatric symptoms in women may be exacerbated during specific phases of the menstrual cycle, particularly the premenstrual phase and the first days of menstruation (Handy et al., 2022). Disorders, often comorbid with ADHD, such as eating disorders, have also been associated with hormonal fluctuations across the menstrual cycle (Edler et al., 2007; Klump et al., 2013). The menstrual cycle starts with the onset of menstruation. This is the beginning of the follicular phase, which lasts until the first day of ovulation and is characterized by gradually increasing estrogen levels. The luteal phase begins at ovulation and lasts until the first day of menstruation. It is typically around 14 days long and characterized by rapidly increasing progesterone and moderately increasing estrogen levels (Diczfalusy & Landren, 1977; Reed & Carr, 2000). The term premenstrual phase, used in this paper, is not a physiologically distinct phase but a descriptive term for the last part of the luteal phase (i.e., the last few days before menstruation). This is the phase when premenstrual symptoms (PMS) most commonly occur.

Of all women of reproductive age, approximately half experience PMS (A et al., 2014), with 3% to 8% meeting the criteria for premenstrual dysphoric disorder (PMDD) (Halbreich et al., 2003; Hantsoo & Epperson, 2015). PMDD is characterized by emotional symptoms such as affective lability, irritability, or anxiety during the luteal phase when progesterone and progesterone metabolites are present. These symptoms are often accompanied by cognitive difficulties such as poor concentration or feeling overwhelmed. Behavioral and somatic changes may also be present, including fatigue, overeating, sleep disturbances, and decreased interest in activities (Reilly et al., 2024). Small studies have shown that more than 40% of women with ADHD exhibit symptoms indicative of PMDD (Dorani et al., 2021).

ADHD has historically been presented as a disorder of externalizing behavior. However, growing awareness of female-specific challenges, has started to shift attention toward the overlap with comorbid conditions and internalization of symptoms, which are more common among females (Young et al., 2020). Females with ADHD and comorbid emotional dysregulation exhibit higher rates of suicidal ideation and attempts, as well as disordered eating and bulimia nervosa (BN) compared to those without ADHD or PMDD (Nobles et al., 2016; Prasad et al., 2021). Mood and emotional dysregulation, although not part of the formal ADHD diagnostic criteria, are widely recognized as central and impairing features in females with ADHD, affecting their everyday life (Bodalski et al., 2023). Thus, PMDD symptoms of executive dysfunction and emotional dysregulation resemble and overlap with characteristics of ADHD.

This study aims to explore the lived experiences of women of reproductive age with ADHD, focusing on gender-specific challenges. Specifically, we aim to investigate how women with ADHD: (a) experience their symptoms and well-being in relation to hormonal fluctuations during the menstrual cycle and contraceptive use, and (b) perceive hormonal and reproductive counseling within the healthcare system.

## Methods

A qualitative design and analysis method was chosen as it allows for in-depth exploration of the personal experiences of women with ADHD (Braun & Clarke, 2014), enabling a more nuanced understanding of the impact of hormonal fluctuations on daily functioning. Qualitative interviews are an important addition to epidemiological studies and randomized control trials, giving women the possibility to express their feelings and experiences in their own words. The findings are reported following the Standards for Reporting Qualitative Research (SRQR) guidelines (O’Brien et al., 2014). The study was conducted in accordance with “Good research practice” guidelines and the principles of the Helsinki declaration. The study was approved by the Swedish Ethical Review Authority (No: 2023-00645-01). Informed written consent was collected from all participants prior to participating in the study.

### Selection and Description of Participants

Based on the study’s objective, a sample size of 10 to 20 participants was considered sufficient to ensure information power (Malterud et al., 2016). To recruit women diagnosed with ADHD, a purposive sampling strategy was employed. Information about the study was shared through the researchers’ social media channels, inviting women to participate. Additionally, advertisements were placed in both inpatient and outpatient healthcare settings, including psychiatric, maternal health, youth, and sexual health clinics across four healthcare regions in Sweden (Uppsala, Stockholm, Gävleborg, and Västra Götaland). Purposive sampling, which involves selecting participants who are rich in information, enables a thorough examination of their experiences (Patton, 2014). The inclusion criteria were: (1) identifying as a woman, (2) having a formal diagnosis of ADHD, and (3) being a biological woman aged 15 years and above of reproductive age. Women who expressed interest in participating were invited consecutively. No formal exclusion criteria were applied, other than being able to participate in Swedish, in order to capture a broad range of experiences.

After having performed 14 interviews, which were all found to be rich in data (Braun & Clarke, 2021), no more interviews were performed guided by the “information-power” concept.

### Data Collection and Measurements

A semi-structured interview guide based on Creswell and Creswell (2017) was developed through an iterative process within the research group. The guide consisted of open-ended questions designed to encourage the women to share and elaborate on their current and previous experiences. After a few background questions, the interview focused on the following three main questions: (1) Have you experienced any problems related to menstruation, the menstrual cycle, or hormonal contraceptives? (2) Have you experienced any variations in daily life functioning (e.g., emotions, cognition, social relations, routines, exercise, sleep, food, and addictive behaviors) during the menstrual cycle? (3) What are your thoughts about the knowledge of professionals within the healthcare system regarding female ADHD (e.g., menstruation, the menstrual cycle, and hormones)? Probing questions were also used to get the women to elaborate on the main themes (e.g., are there any other ways in which you feel variations in your functioning across the menstrual cycle?)

Two pilot interviews were conducted, after which minor adjustments were made to the interview guide, including question order and probing question formulation. The pilot interviews were found to be rich in data and were therefore included in the data analysis. The study was conducted from November 2023 to February 2024. All interviews were performed by authors Julia McTaggart (JM) and Niklas Envall (NE), one-on-one, online via Zoom using end-to-end encryption to ensure confidentiality. The interviews lasted for an average of 64 min, ranging from 30 to 137 min (median: 50.5 min). Transcribed interviews were stored electronically in a password protected research drive at the investigating university.

### Analysis

Data were retrieved from the interview transcripts and analyzed using inductive thematic analysis (Braun & Clarke, 2006). Each transcript was read by three members of the research team to identify patterns and recurring elements in the open-ended responses. These patterns were then categorized into themes and synthesized into key themes related to the research questions of this study

The qualitative analysis consisted of six phases of thematic data analysis as described by Braun and Clarke (2006): (1) Familiarization with the data, (2) Generating initial codes, (3) Searching for themes, (4) Reviewing the themes, (5) Defining and naming the themes, and (6) Producing the report.

### Researcher Characteristics and Reflexivity

Charlotte Borg Skoglund (CBS), Helena Kopp Kallner (HKK), and Lisa Thorell (LT) designed the study and its protocol. CBS and HKK are experienced senior consultants in psychiatry and gynecology respectively, CBS is associate professor (MD, PhD), and HKK is MD and professor in gynecology. They have extensive experience with the patient group and the healthcare system. LT is professor of psychology. NE created the interview guide with input from CBS and JM. NE is a registered nurse midwife, PhD, and senior lecturer with experience from a sexual health clinic. JM is a doctoral student, MD, and a resident in gynecology and obstetrics with experience in clinical work and contraceptive counseling. NE and JM conducted the interviews, and the data analysis was performed by JM, CS, NE, and HKK.

The research group includes a member with an ADHD diagnosis, as well as members with lived experience through close relatives with the condition. In addition, we had an ongoing co-creation process with women with ADHD prior to designing our studies. However, patient representatives were not involved in coding or interpreting the data in the present study.

As researchers with backgrounds in gynecology, midwifery, and psychiatry/psychology, including expertise in ADHD and sexual and reproductive health, we were mindful of our own potential biases throughout the study. To minimize the risk of unintentionally influencing the interview process, interviews were conducted by members of the research team with the least specialized knowledge of ADHD. JM and NE kept a journal to document their thoughts and assumptions during data collection and analysis, ensuring their interpretations were based on the participants’ perspectives. The research team reviewed the transcriptions individually and discussed them multiple times to ensure the data’s authenticity.

JM and NE also considered how cultural, social, and institutional factors influenced the interactions and data collected. By being reflective, we aimed to maintain transparency and rigor in the research. This approach not only improved the study’s trustworthiness but also provided a deeper understanding of the complex dynamics involved.

## Results

A total of 14 interviews with women diagnosed with ADHD were included in the study. The women were aged 22 to 47 years and had been diagnosed with ADHD at ages 15 to 46 years. See Table 1.

**Table 1.** Participant Characteristics.^a^

Characteristic	Mean (*SD*)	Range
Age (years)	33.5 (8.4)	22–47
Age at diagnosis (years)	29.9 (9.0)	15–46
Employment		Proportion *n* (%)
Employed		8 (57)
Self-employed		2 (14)
Student		3 (21)
Marital status		
Married/partnered		8 (57)
Divorced		2 (14)
Single		4 (29)
Children		
Yes		7 (50)
No		7 (50)
Current ADHD medication		
Yes		10 (71)
No		3 (21)
Psychiatric Comorbidity		
Yes		12 (86)
No		2 (14)

a Numbers and proportions do not equal 100% of participants in order to maintain anonymity.

Eight participants were in a stable relationship, defined as either married or in a long-term partnership. Twelve participants reported one or more additional psychiatric diagnoses besides ADHD, with anxiety being the most common comorbid condition (*n* = 8; 57%), followed by depression (*n* = 6; 43%) and eating disorders (*n* = 4; 29%). Nine participants had tried two or more different contraceptive methods, three participants had tried one, and one participant had never tried any contraceptives. Ten participants were currently using ADHD medication.

The qualitative analysis yielded three key themes:

1. Controlled by female hormones
2. Frustration with lack of knowledge/understanding
3. Living with ADHD and comorbidities.

**Figure 1.**
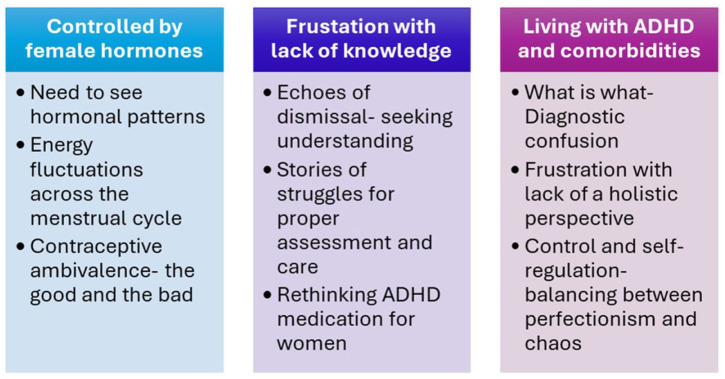
Results of inductive thematic analysis.

Key themes and subthemes are displayed in Figure 1. Illustrative and supportive quotes from the interviews are presented under their related themes below.

### Theme 1: Controlled by Female Hormones

To be controlled by female hormones was a recurring feeling expressed by the participants, who shared a desire to better understand how hormonal fluctuations throughout their menstrual cycles, and in response to contraceptive methods, affect them. This theme includes subthemes related to the participants perceptions of how female hormones influence their emotions, overall wellbeing, and relationships.

#### Need to See Hormonal Patterns

The respondents described feelings of confusion and frustration about not knowing whether their emotions are linked to their menstrual cycle, a consequence of their ADHD symptoms, or part of who they are. Others shared that, over the years, they had gained a deeper understanding of how their cyclical hormones may impact their mood, body image, ADHD symptoms, relationships, daily functioning, feeling of self-worth, and overall quality of life.

P11: To feel down and sad and stuff, and so difficult when you don’t know if it’s part of the cycle or just as it is.

P6: I can feel fat before I get my period and after my period, I feel thin, so it’s a big difference in how I see myself in the different phases of the menstrual cycle.

P9: In this chaos as a teenager. If I just had understood more then. There was so much that happened then, that I couldn’t navigate through. It’s been 15 years and it’s first now that I’m starting to understand what is happening in my body and how it affects my ADHD symptoms.

P5: It has also been very difficult with friends. When I have my menstruation perhaps I become too “all over the place” and a bit too impulsive, so I have had difficulties establishing good relationships. And that has affected me socially. And now that I am older I feel that when I have my menstruation or the week before I become a little . . . like I have no energy to meet people and prefer to be close to those who know me or are close to me.

For some participants, it is the use of period-tracking apps that have helped them notice patterns in their physical and mental well-being related to their menstrual cycle.

P6: My whole life I thought that I had problems with my stomach and now I’ve just understood that it is ovulation and period pain . . . the only thing that helps me see patterns is my period-app.

P4: I think it’s a combination of the insights I get from the period-app and the fact that I now can see a certain regularity - it makes me more aware that a particular phase of my cycle is coming up. That awareness helps me be more forgiving towards myself. If I next week start feeling overwhelmed by everything, maybe it’s not entirely my fault. And that kind of knowledge really makes a difference for my mental well-being.

#### Energy Fluctuations Across the Menstrual Cycle

Many participants reported experiencing challenges and energy fluctuations across different phases of the menstrual cycle and at various stages of life. The time around mid-cycle, most likely the ovulatory phase, was described by several participants as a period of increased energy and motivation, during which many decisions were made and plans created. However, just a few days after these energy surges, the same women reported a lack of energy and an inability to follow through, which they clearly associated with the premenstrual phase of the menstrual cycle. The use of antidepressants to treat premenstrual symptoms was mentioned by a few participants as effective and helpful.

P2: I feel that I get very wired up just before ovulation, it is chaos at that time because I’m trying to do everything at the same time. . . whereas I am pretty stable during my pregnancy.

P4: Every time I have hit the wall or just not managed anything more, it has been right before my period. Every time I quit my job, left the country, broke up from a relationship and so on, it is in the week before.

P13: I sort of have two weeks when everything feels great. I plan and book lots of things, everything runs smoothly, then things become tougher, and I have to take care of everything that I have booked. I have no energy.

P7: I had a period with my ex-husband when we experimented with anti-depressants for PMDS because he thought I had so many tantrums.

#### Contraceptive Ambivalence: The Good and the Bad

Many participants reported having used several contraceptive methods throughout their lives with mixed experiences, including experiencing better emotional regulation with a more stable mood as well as negative mood symptoms with hormonal contraception.

P7: So, I feel this contraceptive injection I have now is good for me. Because my mood is more stable. I don’t have the mood swings that I have during a menstrual cycle.

P2: Ever since I stopped taking hormones and got my regular period back, I feel like it’s just chaos in my brain constantly.

P7: The contraceptive injection I have now feels good for me. It helps stabilize my mood. I don’t experience the mood swings that come with a menstrual cycle. In some way, it also helps me control my mood and emotions.

P4: With the birth control pills . . . I felt more sad. All of my emotions were much more intense.

### Theme 2: Frustration With Lack of Knowledge/Understanding

All participants mentioned the challenges of being a woman with ADHD. They described gender-specific difficulties related to their symptoms, as well as a lack of knowledge and understanding within the healthcare system. Many reported being shuffled between healthcare providers, with no professionals taking a holistic or multidisciplinary approach. Several felt they had to fight hard to get the support they needed, if they ever received it at all.

#### Echoes of Dismissal: Seeking Understanding

Several participants shared experiences of being misunderstood, feeling rejected, or not being taken seriously by healthcare professionals. These encounters often led to a frustrated and defensive attitude and a growing distrust of healthcare resources. Participants also discussed how healthcare providers frequently focused on questions and topics they perceived as irrelevant, rather than addressing hormonal issues and ADHD symptoms.

P8: Not just ignorance, but a terrible lack of interest. And they make you feel like you’re almost crazy . . . I said, what is that? She wrote it down on a post-it note and said I could go home and google it. That was the help I got.

P6: And I said to her, ‘But I would like to do an ADHD assessment.’ And then she was like. . . it was as if she got tired of me and just said, ‘But what do you need an ADHD diagnosis for?

P1: The first time I sought help for my ADHD they just asked me if I have friends . . . and they said that I should lose weight.

#### Stories of Struggles for Proper Assessment and Care

Many participants expressed frustration over the lengthy process of being assessed for and ultimately receiving an ADHD diagnosis. They reflected on whether the lack of knowledge about ADHD in women among healthcare providers, including knowledge about how sex hormones influence ADHD symptoms, may have contributed to delays in assessment, diagnosis, and treatment. One respondent pointed out that despite extensive assessments through several clinics in psychiatry and general medicine, no one had asked about the menstrual cycle.

P13: Nobody realized that it could be ADHD, I had always had so good grades and many of the classic little-boys-who-can’t sit-still-symptoms didn’t fit me . . . If anyone of them had the slightest idea about what ADHD can look like in intelligent women or girls it wouldn’t have, I don’t think it would have, taken 5 or 6 years until . . . (getting a diagnosis)

P14: It is so unfair that women aren’t noticed . . . I’m really happy that I’ve gotten a diagnosis, but that it had to take such a long time doesn’t feel ok.

P2: 500 damn papers and no one asked anything about that (refers to the menstrual cycle).

Several participants expressed a need for more information about gender-specific aspects of ADHD. Amid considerable dissatisfaction among participants regarding the lack of knowledge, there were examples of encounters with healthcare providers who showed genuine interest in learning more about these issues.

P5: I would say that I haven’t experienced getting any good information from a nurse about how ADHD and the menstrual cycle work together or anything like that.

P4: I have shared articles with them, and they have been happy to receive them. So, there are definitely people within healthcare who want there to be more knowledge so that they can share it.

#### Rethinking ADHD Medication for Women

Several women described that they have considered adjusting the dosage of their ADHD medication throughout the menstrual cycle, thinking that it might have different effect depending on which cycle phase they are in. There was a general curiosity about whether it would be possible to optimize or adjust the dosage of ADHD medication as well as medication for comorbid conditions based on hormone status. There were also thoughts about whether hormonal contraceptives could help reduce ADHD symptoms and, if so, potentially serve as an alternative to traditional ADHD medication.

P5: I think it’s a lot of prescribing medication on medication without thinking about that they may work really well when I’m not on my period, but when I have my period I might need extra pills like Atarax and Lergigan (antihistamines with anxiolytic sedative effect) because they think that I have increased anxiety or more depression, but it’s actually ADHD and the menstrual cycle.

P7: It’s often like this, ‘here’s Concerta, go home and see how it feels.’ But for girls, especially those with ADD symptoms, not the hyperactivity, it might be easier for them to take a contraceptive that helps them instead of ADHD medication. Maybe it can help to normalize dopamine levels. Yes, I don’t know. But there might be alternatives instead of just the classic medications.

P6: If I had had more knowledge, I would have liked to change the dose of the ADHD medication throughout the menstrual cycle.

P11: They ask, Are you taking any medication? Yes, and then I say that I take Ritalin and they say okay. And it feels more like a routine question in case you were taking blood thinners or something else, I don’t know. Or something like that. But we have never talked about my ADHD and contraceptives together, never ever.

### Theme 3: Living With ADHD and Comorbidities

Many participants reported one or more psychiatric co-morbidities and shared reflections and challenges related to these conditions. Some suspected they might be on the autism spectrum or had already received a diagnosis. Several women questioned whether their emotional instability might be hormonally driven. Past or current struggles with eating disorders were also common, with some participants noting a perceived worsening of symptoms during the luteal phase.

Participants also expressed that the ambiguity regarding comorbid diagnoses extended to healthcare professionals, who were perceived as lacking a holistic understanding of how ADHD and hormonal fluctuations interact with comorbid diagnoses. This in turn, was seen as contributing to delays in recognition and treatment of both ADHD and other psychiatric comorbidities.

#### What is What?: Diagnostic Confusion

While several participants reported having one or more psychiatric comorbidities, some of them expressed uncertainty about whether these diagnoses were accurate. There was a recurring theme of confusion regarding what was truly causing difficulties in their daily life. If ADHD, hormonal fluctuations, or other underlying conditions best explained their experiences.

P1: I’m quite sure that I have autism, but I don’t show those symptoms much outwards. It’s more inside, so it’s difficult to prove.

P2: I’m one of those typically high achieving women, diagnosed with anxiety and depression, that suddenly just realizes, Ah, is it this (refers to ADHD) that’s wrong with me?

P8: Maybe I don’t actually have borderline; maybe I have an hormonal imbalance that creates this emotional rollercoaster.

P13: Do I not have ADHD? Or have I just made all this up? What is wrong, if it’s not that?

P13: I wasn’t depressed, it wasn’t that. No, in retrospect it should have been so simple for someone to realize then, that it might have been ADHD.

#### Frustration With Lack of a Holistic Perspective

Several women expressed a frustration regarding not receiving adequate answers to their questions concerning ADHD and hormonal fluctuations, not knowing the actual cause of their difficulties or being recommended help within other medical specialties. Participants expressed experiencing a lack of a holistic perspective, as well as a lack of knowledge, on gender-specific aspects of ADHD amongst healthcare professionals.

P3: More cooperation between different healthcare providers. . . There are a lot of comorbidities and it feels like you either have to go to a psychiatrist or a gynecologist. It doesn’t feel like they have. . . yes, it would be good to have more. . . connected knowledge to make it easier. So it’s not like yes, now you have to go to this one, or now you have to go to that one, and. . . yes. It would be nice for a person like myself who can barely book one appointment, to not have to call around and book 70 different appointments

P9: And the health center says, ‘But that’s a side effect of your ADHD medication. Psychiatry should handle that.

P8: But this is like- “oh darling”, I have heard that so many times for healthcare staff that they see me as a little girl their “darling” but it is really that they see me as stupid and belittle me when I ask relevant questions that they have no good answer to and don’t want to get near. It feels horrible to leave a visit feeling like that- you don’t want to go back.

#### Control and Self-Regulation: Balancing Between Perfectionism and Chaos

Participants who had experienced eating disorders described them as arising from a need to regain a sense of control, compensating for the lack of control they felt in other areas of their lives.

P3: I have had problems with binge eating, overeating, bulimia, everything like that . . . and last week, those days (referring to the days before her period), there were several times when I felt a stronger urge to binge eat or felt a need to do so.

P1: I’ve had a lot of mental health issues. A lot of eating disorders. I've realized lately that I think it has to do with my need for control related to feeling lack of control in my life because of my ADHD . . . many things I have been involved in have been about the need for control, control behaviors, or whatever you want to call it.

P4: It (referring to eating disorders) subsided as soon as I realized that I probably had ADHD and that was the underlying problem. Because as soon as I realized that, I understood that I was just trying to solve a problem, and then I realized that I needed to change tools. And it was very easy, it was like night and day when I started reading and understood that binge eating problems are very common. So just the knowledge that it is a tool (ref: for anxiety relief) that many with ADHD use for self-help was enough for me to no longer have a problem with it.

## Discussion

This qualitative study provides insight into the multifaceted and often challenging experiences of women of reproductive age living with ADHD. The three themes identified illustrate the perceived burden associated with the female-specific ADHD symptomatology. Several participants in our study described years of struggling to receive a correct diagnosis and to understand the underlying causes of their difficulties in managing everyday life. This aligns with previous research showing that women are diagnosed later than men following years of massive healthcare utilization for other conditions (Skoglund et al., 2024). Previous research suggests that delayed diagnosis in females may be due to fewer disruptive behaviors and a predominance of symptoms related to comorbid conditions (Fraticelli et al., 2022; Young et al., 2020).

The possible impact of cyclical hormonal fluctuations on daily functioning throughout the menstrual cycle is a recurring theme in several interviews. Several participants reported repeated patterns of increased energy and productivity during the ovulatory phase, followed by periods of low energy and difficulty managing tasks and emotional self-regulation in the premenstrual phase and the first days of menstruation. While these subjective reports do not confirm ovulation or menstrual phase, the findings align with clinical observations and previous research suggesting that women with ADHD may be particularly sensitive to hormonal changes with two high-risk phases; a periovulatory phase, when the estrogen peaks and then drops, associated with hyperactivity and impulsivity and a premenstrual phase that lasts a few days into menstruation associated with increased inattention, depression, irritability, and emotional dysregulation(Eng et al., 2024; Nolan & Hughes, 2022). The first study to explore the prevalence of hormone-related mood disorders in women with ADHD revealed a notably high rate of PMDD symptoms at 45.5% (Dorani et al., 2021) compared to the general population at 3% to 5% (Hantsoo & Epperson, 2015). A more recent study further showed that women with PMDD were more likely to have comorbid ADHD (Lin et al., 2024). These women also reported higher levels of inattention and impulsivity during the postovulatory and mid-luteal phases compared to women without ADHD which highlights the need for evaluating for ADHD when assessing or treating symptoms of inattention and impulsivity in women with PMDD.

The women in this study reported an abundance of gender-related challenges, including perceived cyclic exacerbations of symptoms related to ADHD and other mental health issues. These findings are in line with prior studies and support a growing consensus that hormonal fluctuations may influence mental health symptoms across several diagnostic categories (Eng et al., 2024; Nolan & Hughes, 2022; Peters et al., 2025). A recent review showed clear evidence of symptom exacerbation during the premenstrual phase and first days of menstruation for psychotic disorders, panic disorder, eating disorders, depression, and borderline personality disorder (Nolan & Hughes, 2022). The considerable, and previously established overlap of ADHD with other psychiatric comorbidities (Hartman et al., 2023) is well demonstrated in our study, with many women self-reporting additional conditions such as depression and eating disorders. Our data also indicates that hormonal fluctuations during the menstrual cycle, particularly in the premenstrual phase and the first days of menstruation, may exacerbate symptoms of these comorbidities.

As discussed earlier, adults with ADHD are more prone to seek psychiatric care for treatment of their comorbidities than for their ADHD (Ginsberg et al., 2014). Research found that nearly one-third of adults with eating disorder screened positive for possible ADHD, with the highest rates (35%–37%) in BN and the binge/purge subtype of anorexia nervosa (Svedlund et al., 2017). The risk of being diagnosed with an eating disorder is also increased in an ADHD-population (Nazar et al., 2016). Since women are strongly overrepresented among individuals with eating disorders (Striegel-Moore & Bulik, 2007) and are diagnosed on average 4 years later than men (Skoglund et al., 2024) many cases of ADHD in this group may go undetected.

Women in our study described experiences of loss of control due to their ADHD, sometimes manifested as binge eating, overeating, bulimia, and a sense of regaining control exemplified through restricting food intake. Gaining insight into these patterns helped participants to manage their eating disorders more effectively, suggesting that recognizing ADHD as an underlying factor may play an important role in improving treatment strategies and self-care management for individuals with concurrent ADHD and eating disorders. Symptom fluctuations in BN and binge eating may also relate to menstrual cycle phase (Edler et al., 2007). This association was mentioned by only a small number of participants, underscoring the need to raise awareness of the potential impact of menstrual cycle phases on psychiatric symptoms in women.

The women in this study reported an abundance of gender-related challenges, including perceived cyclic exacerbations of symptoms related to ADHD and other mental health issues. These findings are in line with prior studies and support a growing consensus that hormonal fluctuations may influence mental health symptoms across several diagnostic categories (Eng et al., 2024; Nolan & Hughes, 2022; Peters et al., 2025). A recent review showed clear evidence of symptom exacerbation during the premenstrual phase and first days of menstruation for psychotic disorders, panic disorder, eating disorders, depression, and borderline personality disorder (Nolan & Hughes, 2022). The considerable, and previously established overlap of ADHD with other psychiatric comorbidities (Hartman et al., 2023) is well demonstrated in our study, with many women reporting additional conditions such as depression and eating disorders. Our data also indicates that hormonal fluctuations during the menstrual cycle, particularly in the premenstrual phase and the first days of menstruation, may exacerbate symptoms of these comorbidities.

Several participants described feeling misunderstood and abandoned by healthcare providers, which is in line with studies showing that the knowledge of ADHD, and specifically female ADHD, is often low (Skoglund et al., 2024). A previous qualitative study emphasized that, in addition to a lack of knowledge about ADHD, delayed diagnosis may also be related to gender stereotypes and successful masking behavior among females (Morgan, 2024). The feeling that no health care providers were willing to take on a comprehensive perspective of the different factors that may affect medication strategies was represented in several interviews. The women in our study also emphasized the lack of questions from health care professionals about hormonal influence and treatment, the current life situation, and the role of comorbid symptoms.

Although the results of the present study show that women perceive a lack of knowledge, understanding, and even interest in understanding their needs, it is encouraging to see that the number of research studies on female ADHD is increasing. A review of research advances and future directions for female ADHD was recently published (Kooij et al., 2025). For example, it has been found that pharmacological treatment can successfully be adjusted based on hormonal fluctuation (de Jong et al., 2023) and that a new therapeutic intervention for ADHD targeting the female cyclical pattern could be a valuable addition to more traditional treatments (de Jong et al., 2024). Consistent with the reports from women in our study, the review also highlights the importance of recognizing the overlap between ADHD symptoms and other mental health problems (e.g., anxiety, PMDD, and depression). In summary, the women experienced a lack of a holistic perspective and knowledge on gender-specific aspects amongst healthcare professionals in psychiatry and a lack of knowledge on ADHD and psychiatric comorbidity among providers of reproductive health. Women in this study expressed thoughts about how their lives could have had a different trajectory, had they received more comprehensive information.

Symptoms of ADHD and comorbidities can also be affected by exogenous hormones in hormonal contraceptives (Roberts et al., 2018). Many participants reported having used several contraceptive methods throughout their lives. Women reported both positive effects such as stabilized mood as well as adverse experiences such as negative mood and emotions. Studies have shown that women with ADHD may react differently to hormonal contraception compared to women without ADHD, such as having an increased risk of developing depression during use (Lundin et al., 2023). However, this knowledge is not widely spread among healthcare providers. The perceived mood stabilizing effect of hormonal contraception could possibly be due to its treatment effect on premenstrual dysphoric disorder (Freeman et al., 2012; Ma & Song, 2023). Systemic contraception that causes anovulation eliminates cyclic mood symptoms caused by hormonal fluctuations. For this to happen, correct use of contraception is crucial. Thus, adding hormonal contraception as a treatment strategy for cyclic exacerbations of ADHD symptoms could help create a more stable hormonal environment over the menstrual cycle as well as balancing energy levels and reducing impulsive behavior. Also, use of effective contraception can mitigate reproductive health risks that are increased in women with ADHD, such as unplanned pregnancies, teenage births, premature deliveries and post-partum depression (Andersson et al., 2023; Skoglund et al., 2019). Our findings support the need for healthcare professionals to deepen their understanding of the role that sex hormones play in female ADHD, and the role ADHD plays in reproductive health.

Although the results of the present study show that women perceive a lack of knowledge, understanding, and even interest in understanding their needs, it is encouraging to see that the number of research studies on female ADHD is increasing and that a review of research advances and future directions for female ADHD was recently published (Kooij et al., 2025). For example, it has been found that pharmacological treatment can successfully be adjusted based on hormonal fluctuation (de Jong et al., 2023) and that a new therapeutic intervention for ADHD targeting the female cyclical pattern could be a valuable addition to more traditional treatments (de Jong et al., 2024). Consistent with the reports from women in our study, the review also highlights the importance of recognizing the overlap between ADHD symptoms and other mental health problems (e.g., anxiety, PMDD, and depression). In summary, the women experienced a lack of a holistic perspective and knowledge on gender-specific aspects amongst healthcare professionals in psychiatry and a lack of knowledge on ADHD and psychiatric comorbidity among providers of reproductive health. Women in this study expressed thoughts about how their lives could have had a different trajectory, had they received more comprehensive information.

### Limitations and Future Directions

This is a qualitative study and as such, no conclusions or claims of representativity can be made. Women were asked if they had ever received a formal ADHD diagnosis, as this was an inclusion criterion. No diagnostic interviews were conducted, nor were participants asked for any proof of diagnosis within the present study. We did not ask participants about diagnoses such as PCOS or endometriosis, nor were these conditions used as exclusion criteria. While they may influence experiences of the menstrual cycle, inclusion made it possible to capture a broader range of perspectives. None of the participants brought up or discussed these conditions in relation to their experiences.

This study highlights that women with ADHD believe hormonal fluctuations should be considered when addressing ADHD symptoms and comorbidities, as well as when tailoring and assessing treatment. It is essential to increase the general knowledge about female ADHD and in particular how sex-specific factors, such as hormonal changes during the menstrual cycle and the use of hormonal contraception, affect ADHD symptoms and medication efficacy. Both reproductive health providers and psychiatrists need to be informed to ensure correct diagnosis, reduce the risk for psychiatric and somatic comorbidity, decrease healthcare utilization, and ultimately alleviate suffering for these women.
